# Are trial participants adequately safeguarded by the ethics committees—the Indian scenario?

**DOI:** 10.3389/fphar.2014.00143

**Published:** 2014-06-16

**Authors:** Karan Thakkar, Gauri Billa

**Affiliations:** ^1^Clinical Pharmacologist, formerly affiliated to Department of Pharmacology, Grant Medical College & Sir JJ Group of HospitalsMumbai, India; ^2^Clinical Pharmacologist, formerly affiliated to Department of Pharmacology, Seth GS Medical College & KEM hospitalMumbai, India

**Keywords:** clinical trials, bioethics, post-trial access, clinical ethics committee, conflict of interest

Clinical trials are paramount for the development of medical science and advancement in therapeutics. Mankind has made great strides in medical science, thanks to the ever curious scientific acumen of researchers. But since clinical trials involve human “participants,” their safety, and policies to protect their rights justly take precedence over scientific advancement.

In India, the regulatory agency overseeing and approving clinical trials is the CDSCO, “Central Drugs Standard Control Organization” (Central Drugs Standard Control Organization, 2014). The CDSCO considers and approves only those trials that have been approved by the Ethics Committees, thereby, making the Ethics Committees (EC) the most important link in safeguarding participant's rights and safety. Similarly, even in the USA, the FDA (Food and Drug Administration) and the Department of Health and Human Services have given such powers to the ECs to protect subject rights.

Unfortunately, there have been many instances of unethical trials being conducted in India and globally (SOMO, 2008). One such example is the cervical cancer screening clinical trial conducted in India and sponsored by US National Cancer Institute (NCI) and the Bill and Melinda Gates Foundation. In this trial, two groups of women were offered cervical cancer screening while the third group were not, citing the reason that, in India as a standard of care cervical cancer screening is not routinely undertaken. In the group that was not offered screening, there were 250 deaths due to cervical cancer. The ethics committee failed to identify the issue of withholding the “standard of care” and an inadequate written informed consent process (Srinivasan, 2014; Suba, 2014). Another such example is that of approval of a trial for a fake and dangerous drug by the ECs in the USA (Kutz, 2009; Mundy, 2009).

Recently, the CDSCO has made it mandatory for all the ECs to get registered and also laid down the requirements for registration and functioning of ECs (Suvarnapathaki, 2013). This has ushered in the much needed accountability required for proper functioning of the ECs. It has also stipulated that the Independent ECs can review only Bioavailability and Bioequivalence studies and not clinical trials which can be approved only by the Institutional ECs (ethics committees attached to hospitals) (Suvarnapathaki, 2013). But is this enough to safeguard participant rights? What about the other shortcomings like, inadequate training of members in bioethics, lack of regular monitoring of the clinical trials, non-disclosure of conflict of interest by the EC members and so on.

Studies have shown that the awareness and knowledge of EC members regarding various important bioethics concepts is sub-optimal (Brahme and Mehendale, 2009; Pandiya, 2011; Kadam and Karandikar, 2012; Nadig et al., 2014). This is true despite the fact that most of the members were quite senior and had ample clinical and research experience (Brahme and Mehendale, 2009). To obviate this, accreditation of ECs with organizations like SIDCER Recognition Program and the Accreditation of Human Research Protection Program (AAHRP), ICMR (Indian Council of Medical Research) and FERCI (Forum for Ethics Committee Review in India) is recommended (Kadam and Karandikar, 2012). Also, another important step would be introducing a mandatory ethics checklist and a scoring system, for example, from 1 to 5 (1—adequately addressed to 5—grossly neglected). The ethical concerns that could be incorporated in such a list could include—scientific rationale of the study, equitable and fair selection of subjects, favorable risk benefit ratio, the informed consent process, factors likely to affect the voluntariness, post-trial access, subject insurance and compensation, financial inducement, vulnerable population and so on (Pandiya, 2011; Taylor, 2007). This will ensure adequate consideration of all ethical concerns and the scoring system will ensure an objective decision making as against the current system of voting where the majority decides the fate of a trial.

Reviewing a trial protocol and awarding approval doesn't end the task of the EC. Just as the investigator has to provide regular study updates to the EC, the EC should also take initiative in regular on-site monitoring of ongoing clinical trials to affirm the approval granted on a periodic basis (A Bill further to amend the Drugs and Cosmetics Act, 1940). This is something, although mandatory by law, is lacking (Gogtay et al., 2011; Thomas, 2011; Nadig et al., 2014; Shetty et al., 2014). The recent amendment (2013) in the Drugs and Cosmetics act of 1940 has made it mandatory for the ECs to submit periodic reports of ongoing trials to the CDSCO but to what extent this has been implemented remains doubtful (A Bill further to amend the Drugs and Cosmetics Act, 1940).

Another concern that needs to be addressed is the non-disclosure of conflict of interest by the EC members and the so called, “private hospital-investigator-EC” nexus (Fleck, 2004; Pandiya, 2011; Kadam and Karandikar, 2012; Pereira, 2013). To obviate this it has recently been made mandatory to include at least 50% of government sites for clinical trials (Suvarnapathaki, 2013). Even the law doesn't make it mandatory for the EC members to declare conflict of interest and hence it is seldom done. We have come a long way in regulating clinical trials and improving the functioning of ECs but there still exist gaps that need to be filled.

To summarize,

- There's a need to train EC members in bioethics.
- Mandate accreditation of ECs and a check list based scoring system to review protocols to ensure a comprehensive and fair review wherein all the relevant scientific and ethical issues have been adequately addressed.
- Mandatory regular on-site monitoring and submission of periodic reports.
- Legal liability to declare conflict of interest by the EC members.

## Conflict of interest statement

The authors declare that the research was conducted in the absence of any commercial or financial relationships that could be construed as a potential conflict of interest.
